# Prognostic Value of Histologic Subtype and Treatment Modality for T1a Kidney Cancers^1^

**DOI:** 10.3233/kca-190072

**Published:** 2020-03-30

**Authors:** Michael Siev, Audrey Renson, Hung-Jui Tan, Tracy L. Rose, Stella K. Kang, William C. Huang, Marc A. Bjurlin

**Affiliations:** a Department of Urology, Division of Urologic Oncology, NYU Langone Health, New York, NY, USA; b Department of Clinical Research, NYU Langone Hospital – Brooklyn, Brooklyn, NY, USA; c Department of Urology, Lineberger Comprehensive Cancer Center, Multidisciplinary Genitourinary Oncology, University of North Carolina, Chapel Hill, NC, USA; d Department of Hematology/Oncology, Lineberger Comprehensive Cancer Center, Multidisciplinary Genitourinary Oncology, University of North Carolina, Chapel Hill, NC, USA; e Department of Radiology, NYU Langone Health, New York, NY, USA; f Department of Population Health, NYU Langone Health, New York, NY, USA

**Keywords:** Renal cell carcinoma, T1a, histologic subtype, partial nephrectomy, radical nephrectomy, percutaneous ablation, overall survival

## Abstract

**Introduction:**

To evaluate overall survival (OS) of T1a kidney cancers stratified by histologic subtype and curative treatment including partial nephrectomy (PN), percutaneous ablation (PA), and radical nephrectomy (RN).

**Materials and Methods:**

We queried the National Cancer Data Base (2004–2015) for patients with T1a kidney cancers who were treated surgically. OS was estimated by Kaplan-Meier curves based on histologic subtype and management. Cox proportional regression models were used to determine whether histologic subtypes and management procedure predicted OS.

**Results:**

46,014 T1a kidney cancers met inclusion criteria. Kaplan Meier curves demonstrated differences in OS by treatment for clear cell, papillary, chromophobe, and cystic histologic subtypes (all *p* < 0.001), but no differences for sarcomatoid (*p* = 0.110) or collecting duct (*p* = 0.392) were observed. Adjusted Cox regression showed worse OS for PA than PN among patients with clear cell (HR 1.58, 95%CI [1.44–1.73], papillary RCC (1.53 [1.34–1.75]), and chromophobe RCC (2.19 [1.64–2.91]). OS was worse for RN than PN for clear cell (HR 1.38 [1.28–1.50]) papillary (1.34 [1.16–1.56]) and chromophobe RCC (1.92 [1.43–2.58]). Predictive models using Cox proportional hazards incorporating histology and surgical procedure alone were limited (c-index 0.63) while adding demographics demonstrated fair predictive power for OS (c-index 0.73).

**Conclusions:**

In patients with pathologic T1a RCC, patterns of OS differed by surgery and histologic subtype. Patients receiving PN appears to have better prognosis than both PA and RN. However, the incorporation of histologic subtype and treatment modality into a risk stratification model to predict OS had limited utility compared with variables representing competing risks.

## INTRODUCTION

Renal cell carcinoma (RCC) is a highly heterogeneous tumor in terms of oncologic risk, composed of several histological subtypes, and associated with widely varying patient outcomes [1]. RCC is conventionally assessed according to the International Union Against Cancer (UICC) and the American Joint Committee on Cancer (AJCC) recommendations which categorizes the major RCC histologic subtypes as clear cell, papillary, chromophobe, cystic, collecting duct, and medullary [2]. Clear cell comprises 70–80% of renal cell cancers, followed by papillary with 10–15%, and chromophobe at 3–5%. RCC histology has previously been correlated with prognosis [3–5].

More than two-thirds of all RCCs are less than 4 cm (clinical stage T1a) at the time of diagnosis [6]. Management of T1a RCC includes surgery as well as percutaneous energy-based ablation and observation. Both the American Urologic Association [7] and the European Association of Urology [8], as well as the ASCO [9] endorse partial nephrectomy as the preferred primary treatment while radical nephrectomy can be offered if partial nephrectomy is not feasible. Ablative techniques have generally been reserved for patients who are poor surgical candidates due to limited outcomes data and a higher local recurrence rate [7, 10, 11]. As each histologic subtype of RCC has unique molecular mechanisms and pathologic behavior [12], recent studies have suggested that stratification by histologic subtype could lend prognostic value during treatment [13, 14]. Limited comparative data exist on the overall survival (OS) of T1a kidney cancers managed by these treatment options. Specifically, there is a significant paucity of literature comparing OS by these 3 surgical treatments stratified and RCC histologic subtype. Small volume series of RCC subtypes are limited in their ability to provide information regarding the potential independent prognostic value of histologic subtype. Understanding differences in survival based on histologic subtype and treatment modality may allow for individualized decision making, appropriate pre-operative counseling, and aid in patient selection for clinical trials. Additionally, understanding the impact of histologic subtype on management option outcomes has the potential to influence the utility of pre-treatment kidney cancer biopsy.

Our primary study objective was to evaluate OS of T1a kidney cancers stratified by histologic subtype and management modality. We did not include those cancers managed with surveillance since the impact of histology requires a biopsy, which has not yet been commonly adopted or universally endorsed by guidelines [7–9]. Our secondary objective was to determine the impact of histology and treatment type on predicting overall survival. In order to address the limitations of small single institutions series, we used a large, geographically diverse hospital-based cancer registry-based approach. This approach may allow comparisons of much larger patient samples with greater external validity than institution-based cohorts. In the absence of a large randomized trial, large observational studies may be the best source of information on the comparative effectiveness of management strategies for T1a RCC [15]. This study provides new insight into the survival effects of both intrinsic (histological) and extrinsic (management) factors, and can guide urologic surgeons on their management of T1a kidney cancers and their anticipated outcomes.

## MATERIALS AND METHODS

### Data source and study population

Data were obtained from the National Cancer Database (NCDB) 2004–2015, a national cancer registry established by the Commission on Cancer of the American Cancer Society. The NCDB compiles data from >1500 commission-accredited cancer-programs in the United States and Puerto Rico and captures nearly 70% of all newly diagnosed cancer. Trained abstractors gather data using standardized methodology. This study was waived from IRB review as it did not meet New York University criteria for human subject research due to the use of publicly available, de-identified data and is consistent with federal regulations governing human subject research.

**Fig. 1.**
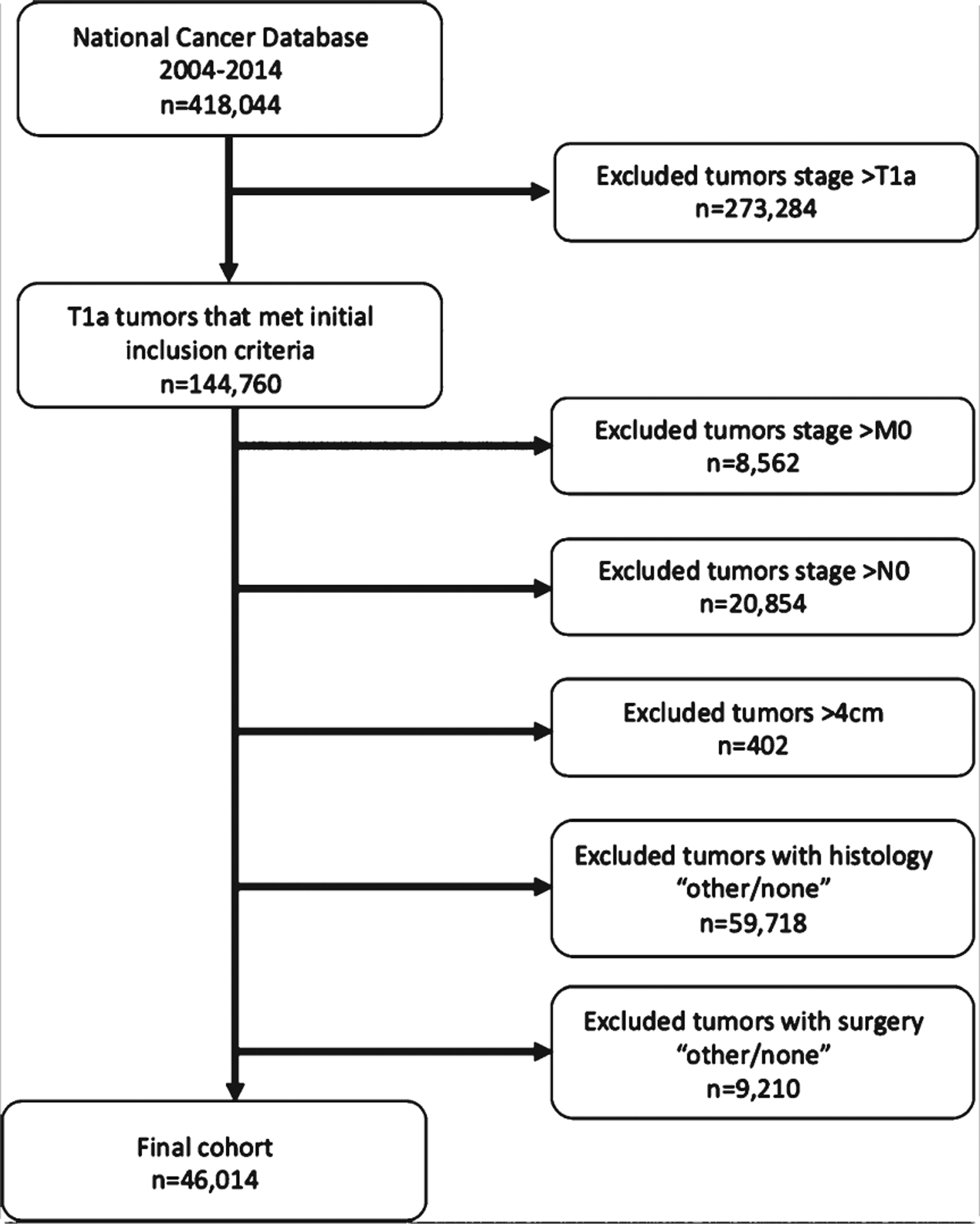
Study flow diagram.

We included T1a kidney cancers, defined as tumor stage c1A or p1A, with malignancy stage cM0 and nodal stage cN0, excluding any tumors with recorded size greater than 4 cm (Fig. 1). We excluded tumors with no histology recorded or with any histology other than the six subtypes defined in this study (see below under Variables). We also excluded patients who did not receive any kidney treatment, the latter including ablation, radical and partial nephrectomy, or whose surgical treatment was marked as “unknown”.

### Variables

The primary outcome was OS in months, defined as the time from diagnosis until death, censored at last contact. The primary predictors were histologic subtype and surgical treatment. We examined six histologic subtypes of renal cancer based on International Classification of Diseases for Oncology, Third Edition (ICD-O-3) codes: clear cell renal cell carcinoma (RCC) (8312), cyst-associated RCC (8316), RCC chromophobe type (8317), RCC sarcomatoid subtype (8318), RCC collecting duct type (8319), and papillary RCC (8260). We examined three surgical treatments based on ICD-O-3 codes: partial nephrectomy [30], radical nephrectomy [40, 50], and ablation [10–14, 21–25].

We also examined several additional demographic and clinical predictors. Demographics included age, sex, race/ethnicity, insurance status, and aggregated census variables for county-level education (proportion with high school diploma in 2000 and 2012), urbanicity in 2003 and 2013 (urban population <20,000 and not adjacent to a metro area, 20,000–250,000 or adjacent to a metro area, 250,000 or more), median income quartiles in 2000 and 2012, and center volume, defined as the number of renal cancer cases reported per year by the facility. Additional clinical predictors included Charlson-Deyo comorbidity score and tumor grade. Tumor grade was defined by NCDB’s tumor grade variable for cases diagnosed prior to 2011, and based on the Collaborative Stage Data Collection System Site-Specific Factor 6 for cases diagnosed in 2011 or later.

### Statistical analysis

To examine differences in OS among histologic and surgical treatment categories, we used Kaplan-Meier survival curves, log-rank tests, and Cox proportional hazards regression. Because there was only one mortality event among collecting duct type tumors receiving ablation, collecting duct type was excluded from Cox models but retained in Kaplan-Meier curves. We first tested a crude Cox model with terms for surgery, histology, and their interaction, the latter of which was tested for significance using a likelihood ratio test.

Because the surgery-by-histology interaction term was highly significant (*p* < 0.0001), we conducted Kaplan-Meier and log rank analyses comparing survival curves between treatments separately within histologies. We also used Cox models to calculate hazard ratios (HRs) for radical nephrectomy and ablation, both compared to partial nephrectomy, within histologies. HRs were estimated both crude and adjusted for age, sex, race/ethnicity, insurance status, census variables, center volume, Charlson-Deyo score, and tumor grade, using standard regression adjustment. There was a relatively small proportion of observations (5.4%, *n* = 2466) with missing data for any covariate therefore we conducted listwise deletion of observations with any missing data in adjusted Cox models.

For continuous variables of age, center volume, and Charlson-Deyo score, we tested for nonlinearity by adding polynomial terms of increasing order in a stepwise manner until Akaike’s Information Criterion was no longer reduced, which resulted in the inclusion of squared and cubed terms for center volume in all analysis using these covariates.

Finally, to explore the prognostic value of histology and surgery, we calculated area under the curve (AUC) of time-dependent receiver operating characteristic curves at each time point, along with Harrell’s C-index, a measure of overall discrimination for survival models. We estimated these parameters for Cox models with terms for histology, surgery, and their interaction only, the additional demographic and clinical predictors only, and histology, surgery, their interaction, and additional predictors. As these were pre-specified models, discrimination statistics were evaluated on the same data used to estimate the models. All analyses were conducted in R software version 3.5.1.

## RESULTS

### Overall survival associated with histologic subtype and treatment modality

A total of 46,014 T1a kidney cancers met inclusion criteria and were included in the analysis. Median age of the cohort was 62 years [IQR 53–71 years], and 37.2% were female. Of note, patients who underwent PA were older (median age 69 [IQR 61–76]) than patients who underwent PN (median age 60 [IQR 52–68]) or RN (median age 62 [IQR 53–71]). 75.7% of patients were white, and 84.7% resided in urban areas. 45.2% of patients were insured with Medicare, 6.0% with Medicaid; 45.3% had private insurance and 3.5% were uninsured. Overall, 92.1% of patients had a Charlson comorbidity index of 0 or 1 : 93.8% of PN, 91.0% of PA and 89.9% of RN patients had a Charlson comorbidity index of 0 or 1.

Of the 46,014 T1a kidney cancers that met inclusion criteria, 52.5% were clear cell, followed by papillary (33.7%), chromophobe (11.5%), cystic (1.8%), sarcomatoid (0.4%) and collecting duct (0.2%). PN was performed in 50%, RN in 32%, and 18% had PA. The overall mortality rate was 15.4% with a median follow up time 51 months [IQR 30–78]. The remaining demographic information can be seen in Table 1.

**Fig. 2.**
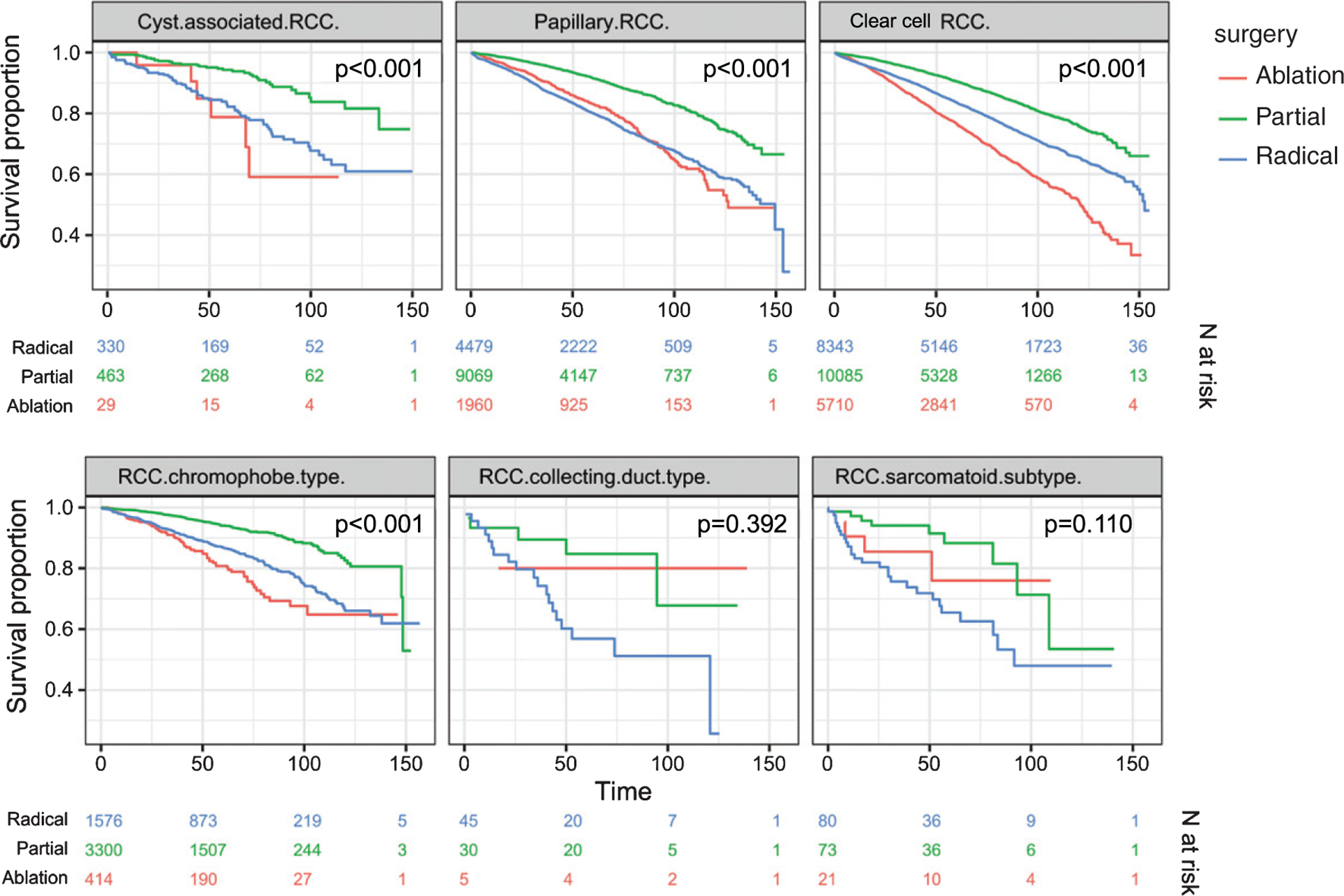
Kaplan-Meier Survival Curves. Adjusted for age, sex, race/ethnicity, insurance status, median income quartile in 2000 and 2012, proportion without high school diploma quartile in 2000 and 2012, urbanicity in 2003 and 2013, Charlson-Deyo index, tumor grade, and facility volume. Key: Green – Partial nephrectomy; Blue – Radical Nephrectomy; Red – Percutaneous ablation.

Kaplan Meier curves demonstrated differences in survival by treatment for clear cell, papillary, chromophobe, and cystic subtypes (all *p* < 0.001), but not for sarcomatoid (*p* = 0.110) or collecting duct (*p* = 0.392) (Fig. 2). Adjusted Cox regression showed worse OS for PA than PN among patients with clear cell (HR 1.58, 95%CI [1.44–1.73], papillary RCC (1.53 [1.45–1.75]), and chromophobe RCC (2.19 [1.64–2.92]). OS was worse for RN than PN for clear cell (HR 1.38 [1.28–1.50]) papillary (1.34 [1.15–1.56]) and chromophobe RCC (1.92 [1.43–2.58]) (Table 2).

### Impact of histology and treatment modality on predictive models

**Fig. 3.**
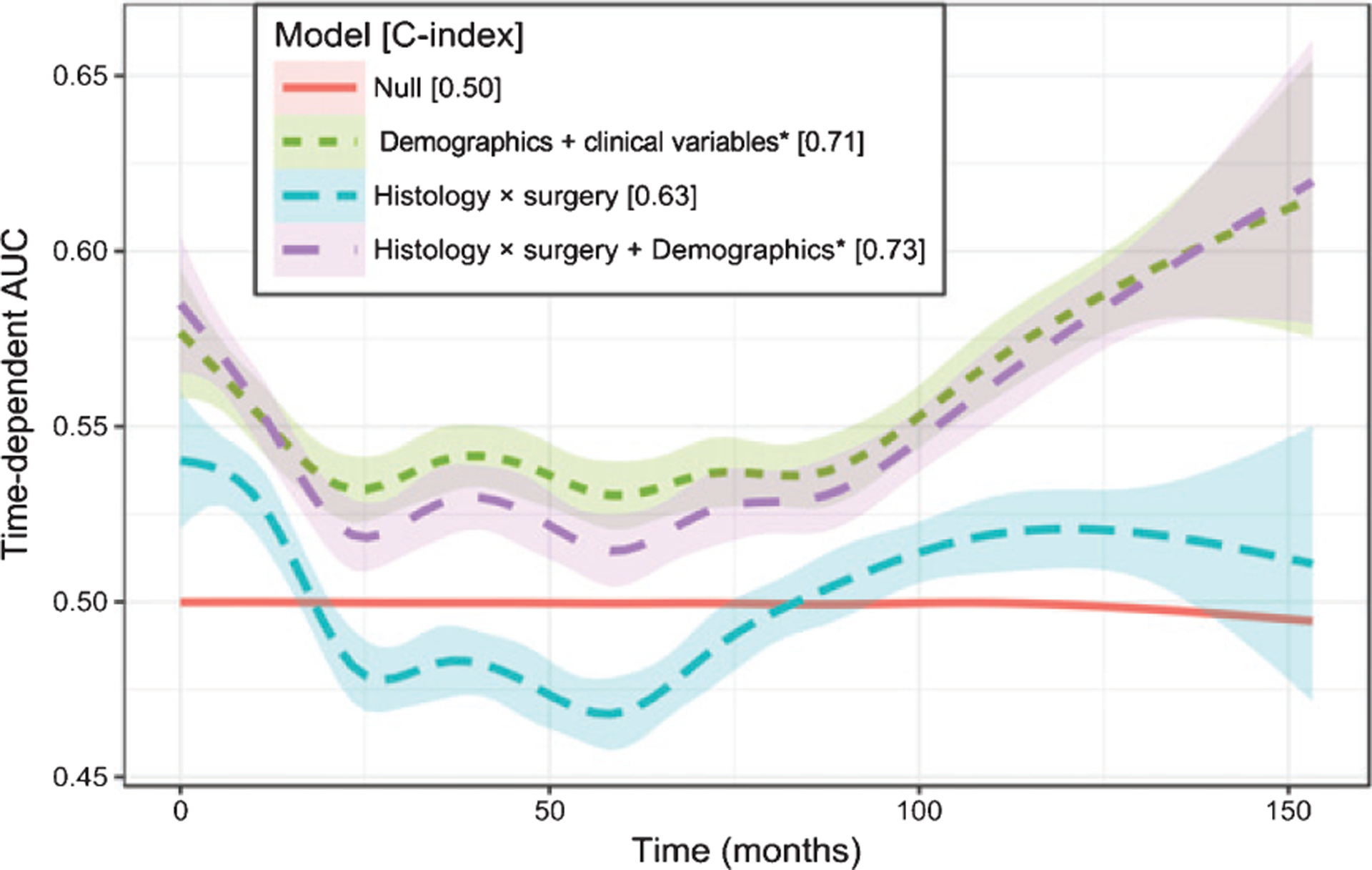
Prediction Performance of Cox Proportional Hazards Models. *Demographics and clinical variables include age, sex, race/ethnicity, insurance status, census median income (2000 and 2012), census proportion without high school diploma (2000 and 2012), urbanicity (2003 and 2013), Charson-Deyo Index, tumor grade, and center volume.

Predictive models using Cox proportional hazards incorporating histology and surgical procedure alone were limited (c-index 0.63) while adding all demographics and clinical variables demonstrated fair predictive power for OS (c-index 0.73). However, a predictive model incorporating demographics and clinical variables alone demonstrated almost equivalent predictive power (c-index 0.71) (Fig. 3).

## DISCUSSION

The ability to accurately predict patient outcomes is an important goal in the management of kidney cancer. Identifying predictive variables allow physicians to fully inform patients about their prognosis and the benefits and harms of each treatment option, which may permit educated decisions about therapy. To date, controversy exists regarding the impact of histological subtype on clinical outcomes of RCC [3–5, 13, 16]. As a primary study objective we sought to evaluate OS of T1a kidney cancers stratified by histologic subtype and treatment modality. We did not include T1a kidney cancers undergoing surveillance in our analysis as tumor histology must be obtained through biopsy which has not yet been widely adopted into guideline management [7–9]. Additionally, renal biopsy is currently employed in <25% of patients with T1a kidney cancers managed non-surgically [17]. In the current analysis of 46,014 patients, our results suggest histologic subtype and surgical management interact to influence overall survival. We observed significant differences in OS of clear cell, papillary, cystic, and chromophobe histologic subtypes of RCC, where patients who underwent PN had higher OS rates for each of these subtypes than those who underwent RN or PA. This effect of treatment modality on OS was not observed with sarcomatoid or collecting duct tumors. These findings may be partly explained by the fact that the incidence of these very aggressive tumors is infrequently found to be <4 cm and because they are often aggressive or symptomatic at the time of diagnosis [16]. Overall, these results support the notion that T1a kidney cancers represent a diverse array of pathologic processes that are grouped together by clinical, not pathological criteria.

Despite the assorted results in the literature on the prognostic impact of RCC histology, RCC represents distinct subtypes with different genetic alterations, morphological appearances, cells of origin, along with varying responses to various surgical management as well as immunotherapy and targeted therapy [1]. We observed these histological subtypes of T1a RCCs to behave in a diverse manner when stratified by surgical management strategy. Although competing risks frequently drive the survival after treatment of T1a kidney cancers, there remains a subset of T1a RCCs where survival is influenced by both histology and treatment modality [18]. This may, in part, be explained by several factors. First, the success of treatment may be impacted by tumor vascularity. Up to 80% of sporadic clear cell RCC loses von Hippel-Lindau function due to a mutation in the short arm of chromosome 3 while neither papillary RCC nor chromophobe RCC have the same genetic alterations. This loss leads to a cascade resulting in the increased expression of vascular endothelial growth factor. Patients with clear cell RCC have been observed to have higher levels of serum vascular endothelial growth factor which augments vascular leakage of tumor cells [19]. Recently, PA of clear cell RCCs have been shown to have less favorable outcomes compared to non-clear cell cancers [20]. Second, achieving negative margins without residual tumor in surgical resection is commonly viewed to be independent of tumor subtype, however, biology of various RCCs may further impact treatment choice and outcomes. Prior studies have shown papillary and clear cell RCC is characterized by a high probability of multifocality (~40%) [21, 22], which is higher than that observed for all patients with RCCs (~5%) [22]. In this regard, the risk for residual satellite tumor may be increased after PN in clear cell and papillary RCC tumors. Third, the presence of a sarcomatoid component within a renal tumor has also been shown to increase the risk of death from RCC, and patients with clear cell RCC or chromophobe RCC are more likely to have a sarcomatoid component than patients with papillary RCC [23].

As a secondary study aim, we determined the impact of histology and surgery type on predicting OS of T1a RCCs. We found that for patients undergoing treatment with curative intent, the additional influence of histology and procedure type on predicting OS was minimal. Specifically, when incorporating histologic subtype and treatment modality into a model which controlled for such as age, sex, race/ethnicity, insurance status, median income, proportion without high school diploma, urbanicity, Charlson-Deyo index, tumor grade, and facility volume, the accuracy of the model to predict OS only incrementally improved. These findings reinforce the notion that RCC histologic subtype and treatment modality of T1a RCCs has limited predictive utility compared with variables representing competing risks [24]. Survival appears to be driven more so by health status and age rather than treatment and pathophysiology, at least at the population-level. Since T1a RCC patients more commonly die of competing factors, OS may appear to be a more pertinent end point rather than cancer specific survival [25]. However, in this setting non-metastatic disease, cancer specific mortality may also provide unique insight into the influence of histology and surgery. Unfortunately, cancer specific mortality is not a variable in the NCDB and cannot be assessed.

Prior studies have established several patient, treatment, and pathologic-level variables that are associated with survival outcomes in RCC subtypes [3, 5, 13, 26]. However, previous studies included all RCC stages and there exists a paucity of data examining stage T1a cancers only. In some series, almost 25% of patients had distant disease and, thus, those studies are not entirely comparable to ours [27]. Furthermore, these studies are not representative of the stage at which most patients currently present. The literature remains discordant regarding the impact of treatment modality stratified by histologic subtype. As a result, current T1a guidelines continue to endorse management strategies based on tumor stage without incorporating RCC histology into decision making [7, 8]. In an analysis of RCC outcomes treated with PN and RN using the Surveillance, Epidemiology, and End Results dataset, Capitanio *et al*. found histologic subtype independently predicted cause-specific mortality, but, similar to our finding, adding histology to a multivariable model failed to improve its accuracy [28].

Our study results have multiple implications for clinical practice. Our data appears to support guidelines endorsing PN for T1a renal masses, regardless of histologic subtype, when the treatment goal is to maximize OS. Although PA more commonly has been reserved for sicker patients who are not operative candidates, we found that the cohort with the highest frequency of Charlson comorbidity indices of 2 or greater were those patients managed with RN. Furthermore, when controlling for clinical and patient demographics including Charlson-Deyo index, PA was associated with a higher HR for mortality than both PN and RN for all histological subtypes, of which clear cell, papillary and chromophobe were significant. These data appear to suggest that patient selection by comorbidities may not be driving the worse outcomes associated with PA. However, we acknowledge that Charlson-Deyo index is limited as a measure of health status. A recent meta-analysis noted an OS benefit in PN vs RN for clinical stage T1a tumors in 10 studies of the SEER cancer database, an effect that was not duplicated in 14 institutional cohort studies. They also noted lower OS for patients undergoing PA as compared to PN or RN [29]. When stratifying by surgical technique, our results suggest PN is associated with moderately better OS compared both PA and RN but this is not a large or precise enough effect size to be useful for prediction in this context.

The current analysis represents an extension of prior studies, with additional patients, extended follow-up and multivariable analysis showing histologic subtype has limited influence on predicting overall survival [28]. These prior study findings, taken together with our results, suggest there is minimal evidence to support the need to identify RCC histologic subtype pre-operatively in order to guide treatment management in terms of OS. This has the potential to influence the utility of pre-treatment kidney cancer biopsy. Outside of determining whether a renal tumor is benign or malignant, it is unknown if and how the knowledge of histologic subtype obtained through biopsy may affect the decision to manage small kidney cancers. Based on our study results, obtaining histologic subtype through a pre-treatment biopsy would not offer any significant improvement in the accuracy to predict OS after treatment. However, there may be a role for knowing the tumor histology even if the model for predicting OS does not change. Biopsies showing less common histologies such as type 2 papillary, HLRCC cancer, and translocation RCC, could influence management strategies such as whether or not to perform a partial vs radical nephrectomy and how wide to resect the tumor. The optimal management strategy for T1a kidney cancers may be driven by patient preferences, competing risks, and treatment complications rather than predicting OS. Our analysis applies only to patients undergoing treatment; the value of a biopsy may lie more in influencing the decision to treat, specifically to rule out benign disease and promote active surveillance for non-aggressive histology.

There are some notable limitations and strengths to our study. Our study was retrospective and possesses the inherent limitations of this type of study design. Data were obtained from the NCDB, a national database based on self-reported data. The limitations inherent in the way the NCDB collects and reports data have been discussed in detail in prior publications [30]. Data from the NCDB is limited by the data being collected by abstractors using ICD codes. Our analysis only applies to those patients who underwent treatment. As this study utilized the NCDB, there was lack of central pathological review. We also had no detailed information about tumor location, a characteristic not available in NCDB but relevant to patient selection. We did not include positive margin rates of upstaging which may have further impacted OS. The location of a tumor within the kidney affects the complexity of PA and PN, and its proximity to other organs may make PA more challenging [31]. We did not have information about all possibly relevant provider characteristics. For example, we did not know which institutions offered PA, nor were we aware of clinician preferences at each institution. To the degree that these characteristics are associated with OS, residual confounding may have been present. Unfortunately, no information on cancer specific mortality is available in the NCDB and cannot be assessed. There may exist small cancer specific mortality differences by histology but this could be overshadowed by competing risks. Given the multiple limitations of the dataset and analysis, we remain cautious in drawing generable conclusions.

Our study is strengthened by its large sample size of over 46,000 tumors and the selection of OS as its primary outcome. To maximize sample size in each treatment group, we did not differentiate between robotic and laparoscopic approaches or among radio frequency, cryo-, and microwave ablation. These ablation methods have some different characteristics. Although combining subtypes of PA and open and minimally invasive surgical approaches within their respective treatment groups may have broadened the variance in outcomes within our treatment groups, this within-group heterogeneity was probably small compared with the differences between the PA, PN, and RN treatment groups.

## CONCLUSIONS

In conclusion, in patients with pathologic T1a RCC, OS differs by histologic subtype. When stratifying by surgical technique, PN is associated with moderately better OS compared both PA and RN but this is not a large or precise enough effect size to be useful for prediction in this context. Incorporating histologic subtype and treatment modality into a risk stratification model to predict OS appears to have limited utility compared with variables representing competing risks. Given the preponderance of T1a kidney cancers, more systematic methods of weighing competing risks are needed for deciding upon optimal surgical management.

## Figures and Tables

**Table 1:** Patient demographics

		Treatment		
	Overall	Partial nephrectomy	Ablation	Radical nephrectomy
Total	46,014	23,021 (50.0%)	8,140 (17.7%)	14,853 (32.3%)
Age, median [IQR]	62 [53–71]	60 [52–68]	69 [61–76]	62 [53–71]
Sex = Female (%)	17,112 (37.2%)	8,408 (36.5%)	2,890 (35.5%)	5,814 (39.1%)
Race/ethnicity (%)				
Hispanic/Latino	2,122 (4.6%)	1,137 (4.9%)	289 (3.6%)	696 (4.7%)
Non-Hispanic Black	7,620 (16.6%)	3,527 (15.3%)	1,114 (13.7%)	2,979 (20.1%)
Non-Hispanic White	34,845 (75.7%)	17,595 (76.4%)	6,497 (79.8%)	10,753 (72.4%)
Other or Unknown	1,427 (3.1%)	762 (3.3%)	240 (2.9%)	425 (2.9%)
Insurance status (%)				
Medicaid / other government	2,774 (6.0%)	1,434 (6.2%)	462 (5.7%)	878 (5.9%)
Medicare	20,793 (45.2%)	8,514 (37.0%)	5,024 (61.7%)	7,255 (48.8%)
Private Insurance/Managed Care	20,845 (45.3%)	12,223 (53.1%)	2,454 (30.1%)	6,168 (41.5%)
Uninsured / unknown	1,602 (3.5%)	850 (3.7%)	200 (2.5%)	552 (3.7%)
Median Income, 2012 (%)				
<$38,000	8,448 (18.5%)	3,907 (17.1%)	1,444 (17.9%)	3,097 (21.1%)
$38,000-$47,999	10,121 (22.2%)	4,842 (21.2%)	1,901 (23.5%)	3,378 (23.0%)
$48,000-$62,999	12,073 (26.4%)	5,913 (25.8%)	2,224 (27.5%)	3,936 (26.8%)
$63,000+	15,022 (32.9%)	8,228 (35.9%)	2,509 (31.1%)	4,285 (29.2%)
Percent with no high school diploma, 2012 (%)				
<7.0%	10,927 (23.9%)	5,783 (25.3%)	1,992 (24.7%)	3,152 (21.4%)
7.0–12.9%	14,728 (32.2%)	7,527 (32.9%)	2,590 (32.1%)	4,611 (31.4%)
13.0–20.9%	12,155 (26.6%)	5,837 (25.5%)	2,182 (27.0%)	4,136 (28.1%)
> = 21%	7,867 (17.2%)	3,748 (16.4%)	1,315 (16.3%)	2,804 (19.1%)
Urbanicity, 2013 (%)				
Rural	2,502 (5.6%)	1,097 (4.9%)	537 (6.8%)	868 (6.0%)
Semi-urban	4,348 (9.7%)	2,088 (9.3%)	780 (9.9%)	1,480 (10.2%)
Urban	37,892 (84.7%)	19,253 (85.8%)	6,541 (83.2%)	12,098 (83.7%)
Charlson Comorbidity Index (%)				
0	32,254 (70.1%)	16,595 (72.1%)	5,602 (68.8%)	10,057 (67.7%)
1	10,109 (22.0%)	5,006 (21.7%)	1,811 (22.2%)	3,292 (22.2%)
2	2,629 (5.7%)	1,077 (4.7%)	530 (6.5%)	1,022 (6.9%)
3	1,022 (2.2%)	343 (1.5%)	197 (2.4%)	482 (3.2%)
Mortality (%)	7,071 (15.4%)	2,048 (8.9%)	1,924 (23.6%)	3,099 (20.9%)
Follow-up time (months), median [IQR]	51 [30–78]	49 [29–74]	49 [28–76]	58 [31–87]
Tumor grade (%)				
I-II	27,338 (59.4%)	15,143 (65.8%)	2,556 (31.4%)	9,639 (64.9%)
III	7,729 (16.8%)	4,413 (19.2%)	245 (3.0%)	3,071 (20.7%)
IV	663 (1.4%)	342 (1.5%)	32 (0.4%)	289 (1.9%)
Other	10,284 (22.3%)	3,123 (13.6%)	5,307 (65.2%)	1,854 (12.5%)
Histology (%)				
RCC clear cell (8312)	24,139 (52.5%)	10,085 (43.8%)	5,711 (70.2%)	8,343 (56.2%)
Cyst associated RCC (8316)	822 (1.8%)	463 (2.0%)	29 (0.4%)	330 (2.2%)
Papillary RCC (8260)	15,508 (33.7%)	9,069 (39.4%)	1,960 (24.1%)	4,479 (30.2%)
RCC chromophobe type (8317)	5,291 (11.5%)	3,301 (14.3%)	414 (5.1%)	1,576 (10.6%)
RCC collecting duct type (8319)	80 (0.2%)	30 (0.1%)	5 (0.1%)	45 (0.3%)
RCC sarcomatoid subtype (8318)	174 (0.4%)	73 (0.3%)	21 (0.3%)	80 (0.5%)

**Table 2:** Cox Proportional Hazards

	Partial nephrectomy				Ablation				Radical nephrectomy			
	HR	95% CI	*p*-value	N events	HR	95% CI	*p*-value	N events	HR	95% CI	*p*-value	N events
RCC (8312)	1	Reference	–	1795	1.58	1.44 to 1.73	<0.001	1491	1.38	1.28 to 1.50	<0.001	1795
Cyst associated RCC (8316)	1	Reference	–	68	2.48	0.97 to 6.32	0.058	6	2.17	0.85 to 5.57	0.106	68
Papillary RCC (8260)	1	Reference	–	946	1.53	1.34 to 1.75	<0.001	350	1.34	1.16 to 1.56	<0.001	946
RCC chromophobe type (8317)	1	Reference	–	244	2.19	1.64 to 2.91	<0.001	72	1.92	1.43 to 2.58	<0.001	244
RCC sarcomatoid subtype (8318)	1	Reference	–	27	0.87	0.27 to 2.83	0.817	4	0.76	0.23 to 2.49	0.654	27

Adjusted hazard ratios for mortality in ablation and partial nephrectomy, vs. radical nephrectomy, stratified^†^ by histologic subtype, using Cox proportional hazards regression (*n* = 43,472). Adjusted for age, sex, race/ethnicity, insurance status, median income quartile in 2000 and 2012, proportion without high school diploma quartile in 2000 and 2012, urbanicity in 2003 and 2013, Charlson-Deyo index, tumor grade, and facility volume.

† Coefficients are stratified by histologic subtype because histology-by-surgery interaction term was significant (*p* < 0.00001).
